# Synthesis of EPA- and DHA-Enriched Structured Acylglycerols at the *sn*-2 Position Starting from Commercial Salmon Oil by Enzymatic Lipase Catalysis under Supercritical Conditions

**DOI:** 10.3390/molecules26113094

**Published:** 2021-05-22

**Authors:** Gretel Dovale-Rosabal, Alicia Rodríguez, Alejandra Espinosa, Andrés Barriga, Santiago P. Aubourg

**Affiliations:** 1 Department of Food Science and Chemical Technology, Faculty of Chemical and Pharmaceutical Sciences, University of Chile, Santos Dumont 964, Santiago 8380494, Chile; gretel.dovale@ug.uchile.cl; 2 Department of Medical Technology, Faculty of Medicine, University of Chile, Santos Dumont 964, Santiago 8380000, Chile; bespinosa@med.uchile.cl; 3 Centre of Studies for the Development of Chemistry (CEPEDEQ), Faculty of Chemical and Pharmaceutical Sciences, University of Chile, Santos Dumont 964, Santiago 8380494, Chile; abarriga@ciq.uchile.cl; 4 Department of Food Technology, Marine Research Institute (CSIC), Eduardo Cabello, 6, 36208 Vigo, Spain

**Keywords:** *n*-3 LCPUFA, EPA/DHA *sn*-2 position, salmon oil, immobilized lipase B, *Candida antarctica* (nonspecific), CO_2_ supercritical extraction, structured acylglycerols, intra–interesterification, MALDI-TOF

## Abstract

There is consistent evidence that long-chain polyunsaturated fatty acids (LCPUFA) belonging to the *n*-3 series, i.e., eicosapentaenoic (20:5*n*-3, EPA) and docosahexaenoic (22:6*n*-3, DHA) acids, decrease the risk of heart, circulatory and inflammatory diseases. Furthermore, the bioavailability of such fatty acids has been shown to depend on their location in triacylglycerol (TG) molecules at the *sn*-2 position. Consequently, great attention has been accorded to the synthesis of structured acylglycerols (sAG), which include EPA or DHA at the *sn*-2 position. The aim of this work was to synthesize sAG starting from deodorized refined commercial salmon oil. For this, immobilized lipase B from *Candida antarctica* (nonspecific) was used as a catalyst for the intra–interesterification process under CO_2_ supercritical conditions (CO_2_SC). According to the CO_2_SC reaction time, three different fractions including sAG compounds were obtained. The location of EPA and DHA at the *sn*-2 position in the resulting glycerol backbone was identified by mass spectrometry (MALDI-TOF) analysis. In all fractions obtained, a marked decrease in the starting TG content was observed, while an increase in the DHA content at the *sn*-2 position was detected. The fraction obtained after the longest reaction time period (2 h) led to the highest yield of *sn*-2 position DHA in the resulting sAG molecule.

## 1. Introduction

There is consistent evidence that *n*-3 long-chain polyunsaturated fatty acids *(n*-3 LCPUFA) produce important health benefits, evidencing their role as mediating bioactive lipids within the protective action exerted by diets rich in these compounds [1]. Marine oils are widely used due to their high content of *n*-3 LCPUFA, such as EPA and DHA, and their consumption reports a low prevalence of heart, circulatory and inflammatory diseases as they reduce the blood pressure in patients with systolic hypertension [2]. A low *n*-6/*n*-3 ratio, together with the daily consumption of *n*-3 LCPUFA, exerts a great influence on the prevention of non-communicable diseases [3], as well as on the decrease in mortality and cardiac risk [4]. The *n*-3 LCPUFA are therefore considered as determining factors in the prevention and treatment of cardiovascular and neurodegenerative diseases [5]. A diet rich in *n*-3 LCPUFA plays an important role in the last trimester of pregnancy and in child nutrition, since DHA especially influences the development and functioning of the nervous system and visual organs of fetuses and newborns [6]. According to the FAO [7], the daily adequate consumption of EPA + DHA should be 250 mg, while in the case of children, daily 150 mg consumption would be necessary for optimal brain development. In addition to the consumption of fish, other products containing *n*-3 LCPUFA are currently used, such as capsules and foods declared safe from a chemical, organoleptic and toxicological point of view.

In the digestive process, an important hydrolysis product of triacylglycerols (TG) by lipases is 2-monoacylglycerol (2-MAG), which is absorbed through the wall of the intestine. The formation of 2-MAG is due to the fact that lipases hydrolyze mainly the *sn*-1 and *sn*-3 positions of the glycerol backbone [8]. Therefore, the specific distribution of fatty acids (FAs) in the glycerol molecule can play a key role in the digestion and absorption of lipids together with the physical state and their phase behavior in water [9]. Therefore, the location of EPA and DHA in the *sn*-2 position is essential to maximize their bioavailability. Commonly, sAG having their *sn*-2 group substituted with a long-chain FA are characterized by their unique nutritional and physiological activity. Notably, the *n*-3 LCPUFA at the *sn*-2 position of TG with medium-chain FAs at the *sn*-1 and *sn*-3 positions are easily absorbed [10]. This is due to the fact that pancreatic lipase at the digestive level releases medium-chain FAs from the extreme positions, which are quickly absorbed and used as an energy source, while 2-MAG is rapidly absorbed in the enterocyte [11]. For this reason, diacylglycerols (DG) with a low energy intake are also considered adequate for the control of obesity and for people with fat malabsorption and other metabolic problems [12].

Structured acylglycerols (sAG) or structured lipids (SL), whose composition and positional distribution of FAs in the glycerol skeleton have been modified, can be obtained by enzymatic catalysis reactions and/or genetic engineering. As a result, “tailor-made” lipids can be obtained with favorable physical characteristics and chemical properties, as well as nutritional benefits [10]. In order to maximize the FA bioavailability, sAG should present a particular FA distribution at the glycerol skeleton [13,14]. Among biocatalysts, lipases have shown to be the most versatile on the basis of their regiospecificity for modifying oils and fats to obtain high-added-value products, such as EPA + DHA supplements, among other sAG. Lipases are defined as enzymes that hydrolyze FAs and alcohol ester bonds and catalyze esterification and transesterification reactions (acidolysis, alcoholysis and interesterification). Additionally, intra–interesterification including interchange of FAs in the same TG molecule backbone can occur. In general, hydrolysis takes place in media with a high water content, esterification takes place in media with a minimal water content and transesterification is efficiently catalyzed in a waterless mixture using immobilized enzymes. Lipases are widely distributed in animals, plants and microorganisms. Those of animal origin are obtained from gastric, intestinal and pancreatic tissues. Vegetable lipases abound in oilseeds (soybeans and peanuts), while microbial ones are produced mainly by fungi and yeasts such as *Aspergillus niger*, *Mucor miehei*, *Rhizopus delemar*, *Geotrichum candidum*, *Candida rugosa* and *Candida antarctica*. Lipases differ in their properties according to the organisms that originate them. The commercial preparations that are used for the modification of oils and fats mostly come from microorganisms [15]. Lipases act on the carboxylic ester bonds present in TG to release FAs and TG. Interestingly, lipases have positional specificity for FAs in TG, which is particularly important in lipid modification [16,17]. Thus, their specificity towards different FAs bound to TG is affected by many factors such as the distribution of FAs in the three positions of the TG molecule [18]. Notably, immobilized lipase B from *Candida antarctica* (CALB) has shown to be nonspecific in the intra–interesterification process of TG, meaning that acyl groups are not introduced in specific positions of the glycerol since the reaction occurs randomly with respect to the position.

The employment of supercritical fluids (FSC) has shown many advantages to produce sAG on the basis that such fluids can be defined as a compressible matter, they behave like gases but have the density of a liquid (0.1–1 g/mL) and, therefore, they have solvent power [19,20,21,22]. Among supercritical fluids, carbon dioxide (CO_2_SC) is found to be an ideal solvent for food applications. It is considered a Generally Recognized as Safe (GRAS) compound [23], has a variable density and great solvent power and can be easily separated from the reaction medium by depressurization. The greatest advantages of CO_2_SC over other liquid solvents are its high diffusivity, and low viscosity and surface tension, which allow it to accelerate mass transfer in enzymatic reactions [20,24]. When CO_2_ is compressed at a temperature of 31.1 °C and a pressure of 72.9 atm above its critical point, it does not liquefy but reaches a dense gaseous state and behaves like a solvent. The use of CO_2_SC for biologically active lipid synthesis significantly reduces the use of organic solvents, avoids waste removal problems, eliminates the use of potentially toxic and flammable solvents and reduces the reaction time. Notably, the use of lipases in CO_2_SC to produce SL is to increase the solubility of lipid and hydrophobic substrates in nonpolar media, producing reverse reactions to hydrolysis and favoring synthesis such as esterification and transesterification [25].

The main aim of the current study was the synthesis of sAG starting from commercial salmon oil. For carrying out the intra–interesterification, immobilized lipase B from *Candida antarctica* (nonspecific) (CALB) Novozyme^R^ 435 under CO_2_SC conditions was used. This commercial enzyme was chosen for being nonspecific, as well as for its avalaiblity and efficiency in this type of process. Furthermore, identification and checking of EPA and DHA presence at the *sn*-2 position during oil fractionation were carried out by mass spectrometry (MS, MALDI-TOF). This analytical technique has shown to be an accurate tool for identifying the FA presence in oils as well as their locations in TG molecules [26,27,28]. The novelty of the current study can be explained on the basis of using a combination of advanced processing conditions and analytical tools to obtain sAG compounds from commercial salmon oil susceptible to be employed in the human diet.

## 2. Results

### 2.1. Physical and Chemical Characterization of Deodorized Refined Commercial Salmon Oil (DRCSO)

Table 1 shows the DRCSO characterization by physical and chemical analyses. Values obtained for the free fatty acids (FFA) content, peroxide value (PV), *p*-anisidine value (AV) and total oxidation (TOTOX) (0.20 ± 0.00 g oleic acid/100 g oil; 0.40 ± 0.00 meq active oxygen/kg oil; 1.13 ± 0.02; and 1.93 ± 0.02, respectively) reveal low lipid oxidation and hydrolytic development, which agrees with previous research on different kinds of fish oils, including salmon oil [29,30,31]. Notably, the present DRCSO oxidation values found are under the limits recommended for human consumption (i.e., 15–20 for the AV value) [32,33,34,35].

The value of FFA indicates the degree of hydrolytic deterioration that the fat has suffered, being a measure of hydrolytic rancidity. The usual values for deteriorated refined fat are between 0.5 and 1.5% [7,36]. The FFA value of DRCSO was very low, in agreement with that obtained by Pando et al. [29], who reported a value of 0.23 ± 0.00 g oleic acid/100 g oil for refined salmon oil. It was also compared with the values obtained in studies previously carried out by Méndez et al. [37], who detected higher values of FFA (3.04 g oleic acid/100 g oil) in studies of oils from industrially farmed salmon. FFA values obtained in the current study comply with the different standards established by world-renowned organizations such as Codex Alimentarius, GOED, EFSA and RSA.

The PV determines the accumulated hydroperoxides corresponding to the first stage of oxidative rancidity, i.e., primary oxidation. The usual values for refined fish oil can attain a maximum of 5 meq active oxygen/kg fat [32,33]. The current value obtained was 0.40 ± 0.00 meq active oxygen/kg oil, lower than the one obtained by Pando et al. [29], who registered 3.54 ± 0.16 meq active oxygen/kg oil in refined salmon oil, thus evidencing the oxidative quality of the current DRCSO.

The AV is a measure of the formation of highly reactive secondary oxidation compounds with a predominance of carbonyl structures such as aldehydes and ketones [38]. For the DRCSO, the AV was lower than the 20-unit limit established by Codex and GOED. In the literature, values of 6.84 ± 0.46, 5.14 ± 1.02 and 3.70 ± 0.33 were observed in refined salmon oil [29,31,39]. In addition, the DRCSO also complies with the Canadian legislation NHP Fish Oil, PhEur Fish Oil Types I and II, Br Pharm, Fish Oil, Australia Natural Fish Oil Types I and II and USP Fish oil [40].

The CD formation correlates with the low PV since they are oxidation indices corresponding to primary oxidation [41]. Therefore, it could be expected that the diene value would be close to zero, as observed in Table 1. Secondary oxidation is represented by the AV and CT indices obtained for the DRCSO, which indicate that the development of such oxidation can be considered as negligible. The results obtained for moisture and impurities are also negligible. It is concluded that the present DRCSO has an optimal initial quality.

### 2.2. Fatty Acid Composition and Quantification of DRCSO

The FA composition for the DRCSO is shown in Table 2 and provided the following decreasing sequence for the most abundant FAs (g/100 g total fatty acids, TFA): 9c-18:1 or oleic acid (36.95 ± 0.08), 9c,12c-18:2 or linoleic acid (15.77 ± 0.07), 16:0 or palmitic acid (12.76 ± 0.03), 9c, 12c, 15c-18:3 or α-linolenic acid (4.91 ± 0.00), EPA (3.92 ± 0.04), DHA (3.83 ± 0.04), 9c-16:1 or palmitoleic acid (3.74 ± 0.01), 18:0 or stearic acid (3.64 ± 0.01), 11c-18:1 or cis-vaccenic acid (3.32 ± 0.00) and, finally, 14:0 or myristic acid (2.90 ± 0.01).

Thus, the analyzed oil reported 20.26% of saturated fatty acids (SFA), 47.05% of monounsaturated fatty acids (MFA) and 32.49% of polyunsaturated fatty acids (PUFA). Concerning the most abundant FAs (oleic, linoleic and palmitic acids), similar results were obtained by Pando et al. [43] and Méndez et al. [37]. However, marked differences were observed for minor FAs by comparing the current results and these previous studies; such differences can be explained on the basis of different culture conditions of the salmon, as they can differ in terms of the diet they have received and the farming sites.

### 2.3. Thin-Layer Chromatography (TLC) of DRCSO

**Figure 1:**
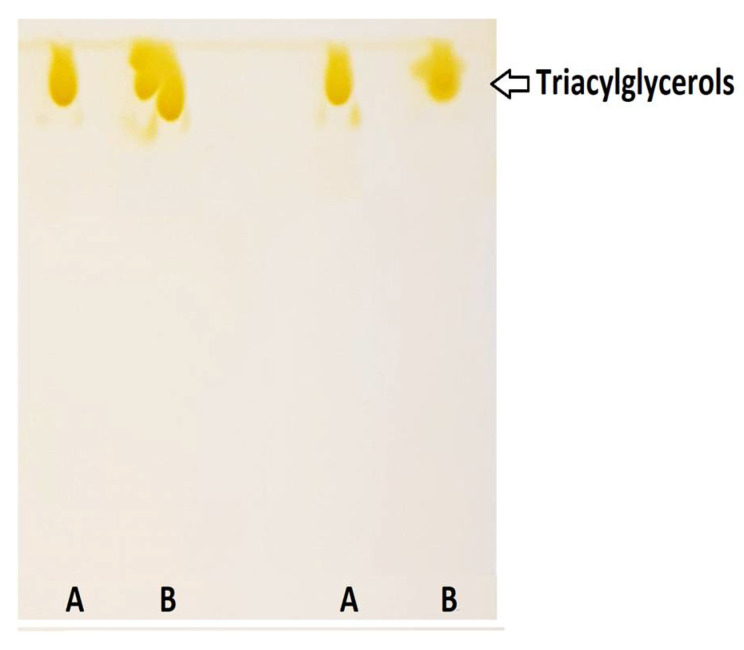
Thin-layer chromatography separation: (**A**) purified DRCSO and (**B**) non-purified DRCSO.

TLC separation of purified DRCSO and non-purified DRCSO is shown in Figure 1. The elution order of the compounds observed showed that FAs are predominantly found in the form of TG in DRCSO. This result is characteristic of refined oils, in which the presence of FAs and DG has been minimized by the refining process and is consistent with the low free acidity value found in this oil.

### 2.4. Position of EPA and DHA in TG of DRCSO Identified in Different Matrices Using Mass Spectrometry (MALDI-TOF)

In Table 3a, the presence of EPA and DHA and their position in DG and TG of DRCSO can be identified with greater probability using the CHCA1 matrix by mass spectrometry. The mass/load range for EPA and DHA was found in the spectrum between 601.4827 and 927.7048 and between 877.7280 and 927.7048 (*m*/*z*), respectively. In this range, it was visualized that EPA and DHA were included in TG, with the exception of DHA, whose spectrum at 601.5 *m*/*z* was found to relate to DG. On the other hand, it was observed that EPA is more likely to be found in the *sn*-2 position, while DHA is mostly located in the *sn*-3 position. These results agree with those found by Berríos et al. [30]; the low probability of DHA in position *sn*-2 may be due to its larger size with respect to EPA.

As it can be observed in Table 3b,c, the presence of EPA and DHA in the monoacylglycerols (MG) and DG of DRCSO was not identified in CMBT1 and DHB1 matrices, respectively. In addition, these matrices only evidenced the probable presence of MG and DG, the appearance of TG being negligible.

Table 3d depicts the position of EPA/DHA in the DG and TG of DRCSO according to identification with the DHB2 matrix. Thus, TG with DHA in different *sn*-3 positions were identified when compared with results obtained from the CHCA1 matrix. In addition, 12 compounds with EPA located at the *sn*-2 position were identified, being the same ones that were visualized in the CHCA1 matrix. As for the CHCA1 matrix, it was visualized that EPA was included in TG in all of this range; on the contrary, DHA was found to relate to DG in the spectrum at a 601.4827 *m*/*z* ratio signal. However, unlike the CHCA1 matrix, a smaller mass/load range was detected for the DHB2 matrix. Thus, EPA and DHA were observed in spectrum values between 601.4827 and 907.7749 and between 905.7593 and 907.7749 *m*/*z*, respectively, thereby indicating a lower identification of TG in the DHB2 matrix compared with the CHCA1 matrix.

Table 3e shows that the CHCA1 matrix contains most of the probable compounds identified when compared with the rest of the matrices. In the DHB2 matrix, 12 probable TG were found that present DHA at the *sn*-3 position, being different from those found in CHCA1. However, due to the importance of the *sn*-2 position, the CHCA1 matrix was chosen as the ideal one to continue the analysis of the samples.

### 2.5. Influence of Fractionation under CO_2_SC Conditions on FA Composition of Structured Acylglycerols (EPA/DHA sAG) during Enzymatic Intra–Interesterification Process of DRCSO

Fractionation was carried out maintaining the process conditions (40 °C and 140 bar) for three periods of time (30, 60 and 120 min). Then, EPA/DHA sAG obtained from the DRCSO were extracted in order to be evaluated by gas liquid chromatography (GLC). The FA composition and quantification of different fractionations of sAG EPA/DHA are shown in Table 4.

The decreasing order in the composition of the FAs of each of the extractions of EPA/DHA, EPA and DHA sAG with respect to DRCSO varied after the enzymatic intra–interesterification process. According to Table 4, the following decreasing FA sequence was obtained for the three most abundant FAs: oleic acid (9c-18:1) > linoleic acid (9c, 12c-18:2) > palmitic acid (16:0). Concerning the remaining FAs, the relative abundance showed to depend on the extraction time. Thus, *cis*-vaccenic acid (7c-18:1) experienced a content increase in the last extractions (60 and 120 min), and myristic acid (14:0), palmitic acid (16:0) and linoleic acid (9c, 12c-18:2) behaved similarly.

The content of EPA and DHA in EPA/DHA sAG also experienced a decrease in the last extraction times, showing significant differences between each of the fractions and with respect to the initial oil (DRCSO) (*p* < 0.05) (Table 4). Thus, the EPA content decreased by 2.6, 6.1 and 8.9% with respect to the DRCSO when fractionation times of 30, 60 and 120 min, respectively, were employed; meanwhile, the DHA content was reduced by 1.0, 18.8 and 32.11%, respectively. According to the current results, the first extraction of fractionation leads to the highest EPA and DHA contents in EPA/DHA sAG starting from DRCSO.

### 2.6. Influence of Fractionation under CO_2_SC Conditions on MG, DG and TG of Purified EPA/DHA sAG Starting from DRCSO during Enzymatic Intra–Interesterification Process of DRCSO

**Figure 2:**
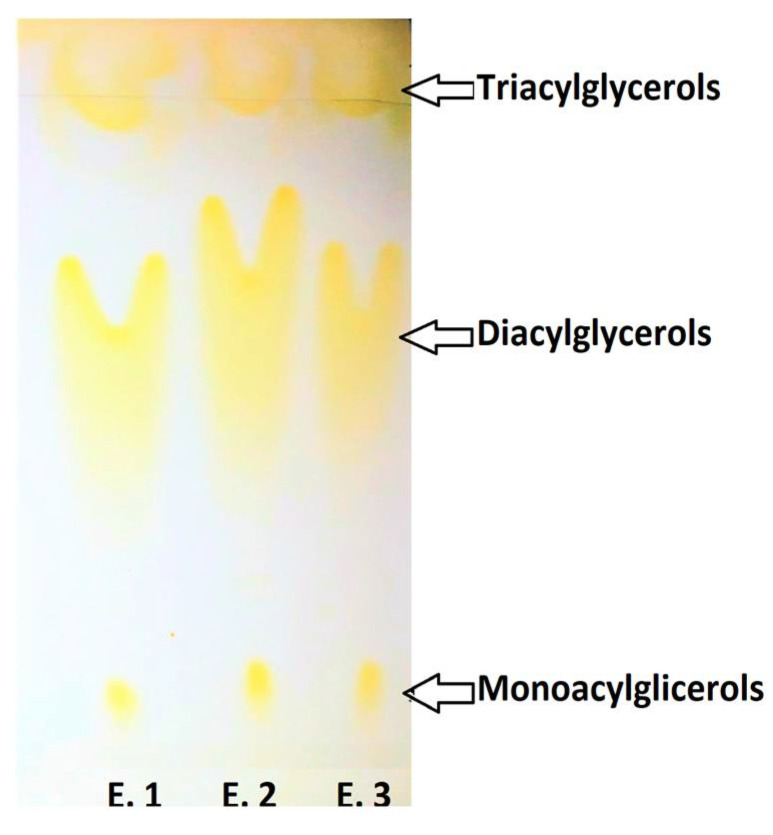
Analysis of monoacylglycerols (MG), diacylglycerols (DG) and triacylglycerols (TG) of purified sAG synthesis by thin-layer chromatography separation. Results obtained by enzymatic intra–interesterification by fractionation under *CO_2_SC* conditions of sAG are shown in different lanes: E. 1 (Extraction or fraction 1), E. 2 (Extraction or fraction 2) and E. 3 (Extraction or fraction 3).

The presence of products resulting from enzymatic intra–interesterification reactions during fractionation under CO_2_SC conditions (i.e., MG, DG and TG of purified EPA/DHA sAG) was observed. The elution of such compounds is observed in Figure 2 according to the polarity degree. As it is nonspecific, the lipase enzyme cuts the bonds that bind FAs to the glycerol structure, being able to rejoin them in a nonspecific way. Consequently, FAs interchange in the same TG molecule backbone, and formation of MG, DG and TG can take place during the enzymatic intra–interesterification process by fractionation under CO_2_SC conditions, as seen in Figure 2.

### 2.7. Influence of Fractionation under CO_2_SC Conditions on the sn-2 Position of EPA and DHA in EPA/DHA sAG during Enzymatic Intra–Interesterification Process of DRSCO

The results in Table 5 mainly show the presence of MG and DG of EPA/DHA sAG determined by mass spectrometry (MALDI-TOF) when the enzymatic intra–interesterification process of DRCSO was performed during the first fractionation under CO_2_SC conditions. As a result, the TG of EPA/DHA sAG could not be identified, showing the total disappearance of these compounds in the fractionated samples when analyzed by mass spectrometry in the CHCA1 matrix. According to the results obtained from Extraction 1 (30 min), only one DG was identified with EPA in position *sn*-2 in the spectrum (namely, 587.4670 *m*/*z*); notably, there was no presence of DHA in MG or DG of EPA/DHA sAG.

**Figure 3:**
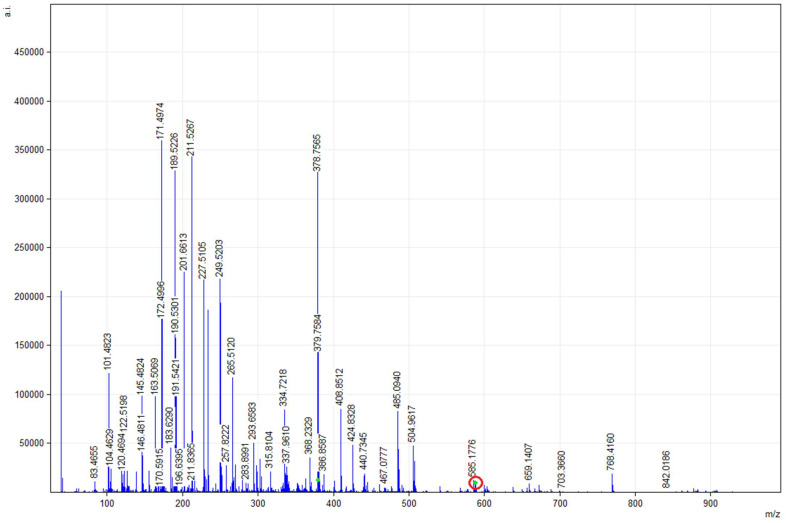
MALDI-TOF mMass report spectrum of glycerolipid (GL) profile resulting from Extraction 1 (30 min). Spectrum obtained between 100 and 900 *m*/*z* ratio values from a sample of sAG mixed with the CHCA1 matrix. The *m*/*z* ratio signal marked in blue corresponds to DG presenting EPA/DHA at the *sn*-2 position. Database: LIPID MAPS for glycerolipids.

Figure 3 shows the MALDI-TOF mMass report spectrum of the glycerolipid (GL) profile resulting from Extraction 1 (30 min) in the CHCA1 matrix. The *m*/*z* signal ratio of 587.4670 (arrow) would correspond to a DG molecule with the following distribution of FA positions according to the mMass report: 14:1 in *sn*-1 position and 5c, 8c, 11c, 14c-20:4 in *sn*-2 position; 12:0 in *sn*-1 position and 7c, 10c, 13c, 16c, 19c-22:5 in *sn*-2 position; 14:0 in *sn*-1 position and 5c, 8c, 11c, 14c, 17c-20:5 in *sn*-2 position; and 18:4 in *sn*-1 position and 16:1 in *sn*-2 position.

The results in Table 6 mainly show the presence of MG and DG of sAG EPA and DHA determined by mass spectrometry (MALDI-TOF) when the enzymatic intra–interesterification process of DRCSO was performed during the second fractionation under CO_2_SC conditions. Concerning Extraction 2 (60 min), the analysis did not show any probability that an EPA or DHA presence could be evidenced in any of the three glycerol positions under the conditions used; additionally, the formation of TG with an EPA/DHA sAG structure was not observed either.

The results in Table 7 depict the presence of MG and DG sAG including EPA and/or DHA detected by mass spectrometry (MALDI-TOF) when the enzymatic intra–interesterification process of DRCSO was performed during the third fractionation under CO_2_SC conditions. In the analysis of Extraction 3 (120 min), the presence of 2-MG and 2-DG with DHA in the *sn*-2 position was identified, a figure higher than that observed in the same matrix (CHCA1) of the initial oil sample, which shows the effect of fractionation during the intra–interesterification process of the last extraction. TG of EPA/DHA sAG were not observed, as in the DRCSO mass spectrometry results, although the presence of MG and DG with EPA in the *sn*-2 position was identified (highlighted in bold).

**Figure 4:**
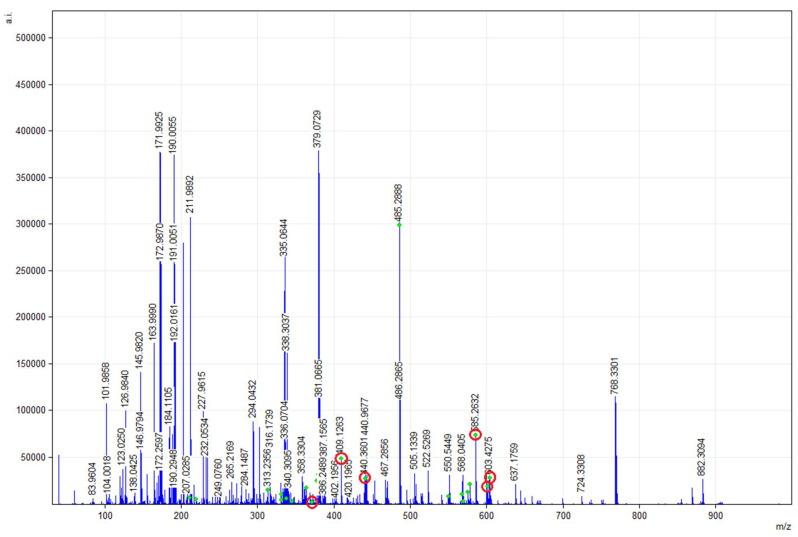
MALDI-TOF mMass report spectrum of glycerolipid (GL) profile of Extraction 3 (120 min). Spectra obtained between 100 and 900 *m*/*z* ratio signals from a sample of sAG mixed with the CHCA1 matrix. Those *m*/*z* ratio signals marked in blue correspond to MG and DG including EPA/DHA at the *sn*-2 position, as well as other MG/DG sAG. Database: LIPID MAPS for glycerolipids.

In Figure 4, the *m*/*z* ratio signal of 377.2686 would correspond to MG of EPA/DHA sAG with EPA in the *sn*-2 position. Meanwhile, the *m*/*z* ratio signals of 585.4514, 599.4670 and 601.4827 would correspond to DG of EPA/DHA sAG with EPA at the *sn*-2 position of its structure. Additionally, the *m*/*z* ratio signals of 409.2925 and 441.2402 would correspond to MG of EPA/DHA sAG with DHA at the *sn*-2 position, and the *m*/*z* ratio signals of 585.4514 and 599.4670 would be related to DG of EPA/DHA sAG with DHA at the *sn*-2 position of its structure according to the mMass report.

## 3. Discussion

In this study, sAG synthesis with EPA and DHA at position *sn*-2 (EPA/DHA sAG) by enzymatic intra–interesterification was prepared starting from DRCSO. The characterization of DRCSO through oxidative stability analyses such as FFA, PV and *p*-AV, among others, showed experimental values within the limits established by RSA, CODEX and GOED; this indicates that the current starting salmon oil is fit to be used. Commercial refined salmon oil evaluated by GLC presented an FA composition characteristic of marine oils. Regarding EPA and DHA, both acids are at the lowest limit of the ranges established for this type of oil with 3.92 ± 0.04 EPA (within a range of 2–6) and 3.83 ± 0.04 DHA (within a range of 3–10) (g/100 g TFA) [32]. This is directly related to the fact that the level of these FAs will depend on the feeding conditions previously applied. One possibility for assessing the diet previously provided is the level of linoleic acid (9c, 12c-18:2) present in the oil; thus, a normal wild individual will have values of 1.5–2.5% of this FA, while cultured individuals can reach values as high as 8–15%. The oil presented 15.77 ± 0.07 g linoleic acid/100 g TFA, which indicates that an important part of the fish’s diet was a source of vegetable oil [44]. This in turn would influence the low level of EPA and DHA, since *n*-3 LCPUFA contents are higher in diets whose energy intake comes from fish oil [45]. Thus, DRCSO presented physicochemical characteristics that allow its use as a raw material for fractionation in a CO_2_SC medium. The effect of the intra–interesterification process under supercritical CO_2_ conditions was also analyzed and was compared with the initial DRCSO. The DRCSO presented EPA in the *sn*-2 position according to the analysis carried out by mass spectrometry. In addition, it was found that the presence of EPA and DHA and their position in the DG and TG of DRCSO can be identified with greater probability using the CHCA1 matrix by mass spectrometry. It was observed that EPA is more likely to be found in the *sn*-2 position, while DHA is mostly located in the *sn*-3 position in TG of DRCSO. The low probability of DHA in position *sn*-2 may be due to the larger size of this LCPUFA with respect to EPA. The EPA/DHA sAG synthesis obtained by intra–interesterification caused changes in the sAG FA composition and content of the fractionated samples obtained with respect to the initial oil when the reaction was catalyzed by the immobilized lipase B enzyme obtained from *Candida antarctica *(Novozyme^R^ 435). The fractionated samples obtained at different times in CO_2_SC media also showed changes in the position of EPA and DHA when analyzed by mass spectrometry (MALDI-TOF). Notably, the third fractionation, whose CO_2_SC conditions were 120 min, 40 °C and 140 bar, presented the highest yield of EPA or DHA in the *sn*-2 position in sAG during the intra–interesterification process.

In addition, the presence of MG and DG with DHA and EPA in position *sn*-2 in EPA/DHA sAG was identified, a figure higher than that observed in the same matrix (CHCA1) of the initial oil sample, which shows the effect of fractionation during the intra–interesterification process of the last extraction. However, MG, DG and TG of EPA/DHA sAG in Extraction 2 (60 min, 40 °C and 140 bar) in the CHCA1 matrix could not be identified, showing a total disappearance of these compounds in the fractionated samples when analyzed by mass spectrometry. Thus, the mass spectrometry results did not show any probability of TG formation being EPA/DHA sAG compounds formed under the extraction conditions used, although the contrary was observed in the TLC results. However, this may be due to the efficiency of the matrix used for the SL. Thus, they show a different behavior from that observed in the initial oil, according to the fact that visualization of the compounds requires the use of a matrix, which would be one of the factors that favor the fragmentation of the compounds [14,46]. Additionally, the ionization process, although corresponding to a soft type, can induce the fragmentation of TG to originate DG or FFA, artificially increasing the content of the samples [47,48]; this fact would explain the difference between values observed in TLC of EPA/DHA sAG and Extraction 3. However, the possibility of greater compounding with DHA and EPA in the *sn*-2 position is an advantage with respect to the initial oil, since it shows that the extraction conditions in a supercritical fluid (temperature, time and pressure) can be manipulated to exert changes in the positioning of EPA and DHA.

## 4. Materials and Methods

Deodorized and refined commercial salmon oil (DRCSO) was provided by Fiordo Austral S. A. (Santiago, Chile). This oil corresponds to the production line authorized to export to the European Community in accordance with the current regulations of the program of quality assurance for human consumption of SERNAPESCA based on NCh 2861:2004. Fatty acid methyl ester (FAME) standards, fatty acid (FA) standards and C23:0-methyl ester (2COT N-23M-A29-4 NU-CHECK-PREP-INC) were purchased from NU-CHEK PREP, INC (Elysian, MN, USA). All solvents and chemicals used (including urea, ethanol, α-tocopherol and n-hexane) were of analytical grade (Merck, Santiago, Chile). DRCSO was stored at −70 °C under nitrogen atmosphere until being employed.

### 4.1. DRCSO Characterization

DRCSO was characterized by means of different chemical analyses (Table 1). The standard AOCS official method [49] procedure was employed for the following assessments: free fatty acids (FFA) content (official method Ca 5a-40), peroxide value (PV; official method Cd 8b-90), *p*-anisidine value (AV; official method Cd 18–19), TOTOX value (official method Cg 3–91), insoluble impurities content (official method Ca 3a-46), unsaponifiable matter (UM) content (official method Ca 6b-53) and moisture and volatile matter contents (official method Ca 2d-25). Additionally, conjugated diene (CD) and triene (CT) formation was measured at 233 nm and 268 nm, respectively [50], the results being expressed in agreement with the following formula: CD (or CT) = B × V/w, where B is the absorbance reading at 233 (or 268) nm, V is the volume (mL) and w is the mass (mg) of oil measured.

### 4.2. DRCSO Fatty Acid Composition

The DRCSO FA profile and EPA/DHA quantification were assessed in an HP 5890 series II GLC with a flame ionization detector (FID) with the injection system split. A fused silica capillary column (100 m length × 0.25 mm × 0.2 μm film thickness) coated with SPTM-2560 (Supelco, Bellefonte, PA, USA) was used. DataApex ClarityTM software (DataApex Ltd., Prague, Czech Republic) for chromatogram analysis was applied. A methylation process was performed to obtain FAMEs. For this, a two-step process was performed according to previous research. The reference standard NU-CHEK GLC463 was used to identify the FA profiles by comparing the retention times [51]. The integration of the chromatographic peaks was carried out from baseline to baseline. The concentration of the different FAMEs was determined from the calibration curves by assessment of the peak/area ratio. The quantification of all the individual FAs (g/100 g TFA) was achieved by employing C23:0 methyl ester as the internal standard according to the AOCS method [52].

### 4.3. Preparation of EPA/DHA sAG by Enzymatic Intra–Interesterification Process under CO_2_-Supercritical Conditions (CO_2_SC) Using Different Extraction Times

EPA/DHA sAG synthesis by enzymatic intra–interesterification was prepared starting from DRCSO. For the enzymatic intra–interesterification, a supercritical CO_2_ reactor Speed SFE system model 7071 (Applied Separation, Allentown, PA, USA) was used, with the following conditions: 20 mL of substrate; supercritical temperature of 40 °C; times of 30, 60 and 120 min; and supercritical pressure of 140 bar. The immobilized lipase B from *Candida antarctica* Novozyme^R^ 435 was employed for the current study for being nonspecific and for its availability and efficiency. This enzyme was maintained at 20% of the substrate [22,24,53,54]. According to the reaction time employed, three different fractions were obtained.

### 4.4. Purification of EPA/DHA sAG by Neutralization with NaOH

The EPA/DHA sAG obtained by the enzymatic intra–interesterification process under CO_2_SC were purified by FA neutralization with NaOH to remove the remnant FFA of the reaction and then collected in hexane for GLC analysis. For this, mixtures with ethanol and phenolphthalein, titration with sodium hydroxide and washes with hexane were carried out. The purification state of each sample was followed by TLC.

### 4.5. Fatty Acid Composition and Quantification of Fractionated EPA/DHA sAG

Fractionation was carried out maintaining the process conditions (40 °C and 140 bar) for three periods of time (30, 60 and 120 min), and the EPA/DHA sAG samples were extracted to be evaluated by GLC, similar to Section 4.2.

### 4.6. Identification of EPA/DHA sAG and DRCSO by TLC

EPA/DHA sAG samples and DRCSO were identified by TLC on Silica gel 60 F254 plates (Merck, Santiago, Chile). A solution of hexane, diethyl ether and glacial acetic acid (80/20/2, *v*/*v*/*v*, respectively) was used as eluent. The order of elution on the chromatographic plate from bottom to top was evaluated according to the compounds’ decreasing polarity: MG, DG and TG [55]. The plates were stained with iodine solution so that unsaturated fatty acids may be visualized.

### 4.7. Positional Analysis of EPA and DHA by Mass Spectrometry (MALDI-TOF)

The location of EPA and DHA in the resulting glycerol backbone was detected by mass spectrometry (MALDI-TOF) analysis. For the determination of EPA or DHA in the *sn*-2 position of the EPA/DHA sAG, samples were analyzed before and after fractionation. After purification, aliquots were taken to be analyzed by mass spectrometry in a “Matrix-Assisted Laser Desorption/Ionization-Time-Of-Flight” (MALDI-TOF) Microflex (Bruker Daltonics Inc., Billerica, MA, USA) instrument in positive ion mode by reflection detection. For the analysis of the spectra, the mMass version 5.5.0 program was used according to the protocols of Strohalm et al. [26], Strohalm et al. [27] and Niedermeyer and Strohalm [28].

Working solutions of the oils with a concentration of 1.0 mg/mL were prepared in chloroform/isopropanol 1:1. The 5-chloro-2-mercaptobenzothiazole (CMBT1) matrix was prepared at a concentration of 10.0 mg/mL in methanol. The working solutions and the matrix were mixed in a 1:1 ratio; then, 0.7 μL was deposited on a micro-scout sample plate (Bruker Daltonics Inc., Billerica, MA, USA). For the detection of the monoisotopic *m*/*z* signals of the acquired spectra, the MALDI-TOF peptides algorithm was used (signal/noise ratio of 3.0 and a relative intensity limit of 0.1%). For the identification, the detected monoisotopic *m*/*z* signals were analyzed and assigned through the Match & Annotate option of the Compound Search tool by comparing them with the theoretical monoisotopic masses of different types of glycerolipids (GL) contained in the base of LIPID MAPS (data version 11/16/2013). Subsequently, the coincidences observed were examined manually, and in each case, the experimental isotope distribution was compared with the theoretical one through the Show Isotopic Pattern option. The same procedure was performed by mixing the samples with the α-cyano-4-hydroxycynamic acid (CHCA1), 2,5-dihydroxybenzoic acid in methanol (DHB1) and 2,5-dihydroxybenzoic acid in methanol and trifluoroacetic acid 0.10% *v*/*v* (DHB2) matrices.

### 4.8. Statistical Analysis

An ANOVA of the parameters with a significance level of *p* ≤ 0.05 was performed according to Fisher’s method (LSD). The 95% confidence intervals of each quality parameter were calculated, considering the number of replicates and the standard deviation of each sample. The statistical program Statgraphics Centurion XVI-2011 (Stat Point Technologies, Inc., Rockville, MD, USA) was used.

## 5. Conclusions

Synthesis of structured acylglycerols including EPA and DHA at the *sn*-2 location by enzymatic intra–interesterification under CO_2_SC from DRCSO was obtained. According to the reaction time employed, three different fractions were obtained, and the location of EPA and DHA in the resulting glycerol backbone was detected by MALDI-TOF analysis. In all fractions obtained, a marked reduction in the starting TG was observed, while a substantial increase in the DHA content at such position was implied. According to the mass spectrometry (MALDI-TOF) analysis, the greatest probability of obtaining an EPA and/or DHA presence in the sn-2 position in DG and TG was identified by the CHCA1 matrix. It was observed that EPA is more likely to be found in the *sn*-2 position, while DHA is mostly located in the *sn*-3 position in TG of DRCSO. The fraction obtained after the longest reaction time (2 h) led to the highest yield of *sn*-2 position DHA in the resulting sAG molecule with respect to DRCSO. Using this method, the analysis by MALDI-TOF established that the current condition in the sAG included the highest levels of EPA or DHA content in the *sn*-2 position of the identified DG. Convenient conditions were established for the intra–interesterification of DRCSO in a fractionation enzymatic reaction to produce acylglycerols with a high biological value that can be employed in future applications of the current study as a potential product for the prevention of the development of different kinds of illnesses such as cardiac, circulatory and inflammatory diseases, adding even more value to synthesized acylglycerols. The current study represents a promising approach in the search for sAG compounds including EPA and/or DHA at the sn-2 position starting from commercial salmon oil.

## Figures and Tables

**Table 1:** Physical and chemical characterization of deodorized refined commercial salmon oil.

Analyses	Value
Free fatty acids (FFA) content (g oleic acid/100 g oil)	0.20 ± 0.00
Peroxide value (PV) (meq active oxygen/kg oil)	0.40 ± 0.00
*p*-anisidine value (AV)	1.13 ± 0.02
TOTOX value	1.93 ± 0.02
Insoluble impurities content (%)	0.00 ± 0.00
Moisture and volatile matter content (%)	0.04 ± 0.00

**Table 2:** Fatty acid (FA) composition and quantification of deodorized commercial refined salmon oil (DRCSO).

Systematic Name	Systematic Abbreviation *	DRCSO (g/100 g TFA)
Lauric acid	12:0	0.06 ± 0.00
Myristic acid	14:0	2.90 ± 0.01
Palmitic acid	16:0	12.76 ± 0.03
*trans*-Palmitoleic acid	9t-16:1	0.07± 0.00
Palmitoleic acid	9c-16:1	3.74 ± 0.01
Heptadecanoic acid	17:0	0.22 ± 0.00
Heptadecenoic acid	10c-17:1	0.13 ± 0.00
Stearic acid	18:0	3.64 ± 0.01
Oleic acid	9c-18:1	36.95 ± 0.08
*cis*-Vaccenic acid	7c-18:1	3.32 ± 0.00
*trans*-Linoelaidic acid	9t, 12t-18:2	0.06 ± 0.00
Linoleic acid	9c, 12c-18:2	15.77 ± 0.07
Arachidic acid	20:0	0.32 ± 0.00
Gamma linolenic acid	6c, 9c, 12c-18:3	0.22 ± 0.00
8-Eicosanoic acid	8c-20:1	0.44 ± 0.01
11-Eicosenoic acid	11c-20:1	1.84 ± 0.04
α-Linolenic acid	9c, 12c, 15c-18:3	4.91 ± 0.00
Eicosadienoic acid	11c, 14c-20:2	1.34 ± 0.01
Behenoic acid	22:0	0.36 ± 0.00
Eicosatrienoic acid	11c,14c,17c-20:3	0.33 ± 0.00
Erucic acid	13c-22:1	0.29 ± 0.01
Arachidonic acid	5c, 8c, 11c, 14c-20:4	0.35 ± 0.04
Docosadienoic acid	13c, 16c-22:2	0.12 ± 0.01
Eicosapentaenoic acid	5c, 8c, 11c, 14c, 17c-20:5	3.92 ± 0.04
Nervonic acid	9c-24:1	0.27 ± 0.03
Docosatetraenoic acid	7c, 10c, 13c, 16c-22:4	0.15 ± 0.03
Docosapentaenoic acid	7c, 10c, 13c, 16c, 19c-22:5	1.68 ± 0.05
Docosahexaenoic acid	4c, 7c, 10c, 13c, 16c, 19c-22:6	3.83 ± 0.04

* Nomenclature of International Union of Pure and Applied Chemistry (IUPAC) [42].

**Table 3:** (**a**) *sn*-2 position of EPA/DHA in the DG and TG of DRCSO identified with the CHCA1 matrix *; (**b**) position of FAs in the MG of DRCSO identified with the CMBT1 matrix *; (**c**) position of FAs in the DG of DRCSO identified with the DHB1 matrix *; (**d**) *sn*-2 position of EPA/DHA in the DG and TG of DRCSO identified with the DHB2 matrix *; (**e**) summary of the presence of EPA/DHA in the DG/TG of DRCSO identified in the different matrices *.

(**a**)				
***m***/***z***	*sn*-2 Position of EPA and/or DHA Identified in CHCA1 Matrix *			
601.4827	DG (15:0/**20:5**/‒)			
877.7280	TG (12:0/**20:5**/22:2), TG (16:0/16:1/**22:6**)			
877.6892	TG (12:0/**20:5**/22:5), TG (14:0/**20:5**/**20:5**), TG (14:1/18:3/**22:6**)			
879.7436	TG (12:0/**20:5**/22:1), TG (16:0/16:0/**22:6**)			
879.6473	TG (13:0/**20:5**/**20:5**)			
879.7048	TG (12:0/**20:5**/22:4), TG (14:0/18:3/**22:6**)			
881.7205	TG (12:0/**20:5**/22:3), TG (14:0/18:2/**22:6**)			
881.6056	TG (12:0/**20:5**/**20:5**)			
881.7593	TG (12:0/**20:5**/22:0), TG (13:0/**20:5**/21:0)			
901.7831	TG (14:0/**20:5**/21:0), TG (13:0/**20:5**/22:0)			
901.6892	TG (12:0/**22:6**/**22:6**), TG (14:1/**20:5**/**22:6**)			
901.728	TG (14:0/**20:5**/22:4), TG (14:1/**20:5**/22:3)			
901.7256	TG (12:0/**20:5**/22:1), TG (14:0/18:0/**22:6**)			
903.7412	TG (12:0/**20:5**/22:0), TG (13:0/**20:5**/21:0)			
903.7436	TG (14:0/**20:5**/22:3), TG (14:1/**20:5**/22:2)			
903.7048	TG (14:1/**20:5**/22:5), TG (16:1/**20:5**/**20:5**), TG (14:0/**20:5**/**22:6**)			
905.7205	TG (14:0/**20:5**/22:5), TG (16:0/**20:5**/**20:5**), TG (14:1/**20:5**/22:4)			
905.7593	TG (14:0/**20:5**/22:2), TG (14:1/**20:5**/22:1)			
905.6995	TG (12:0/**20:5**/21:0)			
905.663	TG (13:0/**20:5**/**22:6**), TG (15:1/**20:5**/**20:5**)			
907.7749	TG (15:1/**20:5**/21:0), TG (14:0/**20:5**/22:1), TG (14:1/**20:5**/22:0)			
907.6786	TG (15:0/**20:5**/**20:5**), TG (13:0/**20:5**/22:5)			
907.7361	TG (14:0/**20:5**/22:4), TG (14:1/**20:5**/22:3)			
907.6212	TG (14:1/**20:5**/**20:5**), TG (12:0/**20:5**/**22:6**)			
927.7987	TG (15:0/**20:5**/22:1), TG (15:1/**20:5**/22:0), TG (16:1/**20:5**/21:0)			
927.7412	TG (14:0/**20:5**/22:2), TG (14:1/**20:5**/22:1)			
927.6838	TG (13:0/**20:5**/22:3)			
927.7436	TG (18:0/**20:5**/**20:5**), TG (16:0/**20:5**/22:5), TG (16:1/**20:5**/22:4)			
927.7048	TG (18:3/**20:5**/**20:5**), TG (18:3/**20:5**/**20:5**), TG (14:1/**22:6**/**22:6**)			
(**b**)				
***m*** **/*z***	FAs Identified in CMBT1 Matrix *			
369.2402	MG (16:0/‒/‒)			
381.2975	MG (18:0/‒/‒)			
401.2662	MG (‒/20:4/‒)			
(**c**)				
***m*** **/*z***	DG Identified in DHB1 Matrix *			
603.4288	DG (14:0/20:0/‒), DG (17:0/17:0/‒), DG (18:0/16:0/‒), DG (16:0/18:0/‒), DG (12:0/22:0/‒)			
603.4288	DG (‒/16:0/18:1)			
603.4288	DG (14:0/18:2/‒), DG (16:1/16:1/‒), DG (14:1/18:1/‒), DG (12:0/20:2/0:0)			
(**d**)				
***m*** **/*z***	***sn*** -2 Position of EPA and/or DHA Identified in DHB2 Matrix *			
601.4827	DG (15:0/**20:5**/‒)			
905.7593	TG (14:0/**20:5**/22:2), TG (14:1/**20:5**/22:1), TG (14:0/20:1/**22:6**), TG (14:1/20:0/**22:6**)			
905.7593	TG (16:0/18:1/**22:6**), TG (16:1/18:0/**22:6**)			
905.6995	TG (12:0/**20:5**/21:0)			
905.7205	TG (14:0/**20:5**/22:5), TG (16:0/**20:5**/**20:5**), TG (14:1/**20:5**/22:4), TG (16:1/18:3/**22:6**)			
905.7205	TG (16:0/18:4/**22:6**), TG (16:1/18:3/**22:6**), TG (14:1/20:3/**22:6**), TG (14:0/20:4/**22:6**)			
907.7361	TG (14:0/**20:5**/22:4), TG (14:1/**20:5**/22:3), TG (16:0/18:3/**22:6**), TG (16:1/18:2/**22:6**)			
907.7749	TG (15:1/**20:5**/21:0), TG (14:0/**20:5**/22:1), TG (14:1/**20:5**/22:0), TG (14:0/20:0/**22:6**)			
(**e**)				
Matrix *	**EPA (*sn*-2)**	**DHA (*sn*-2)**	**DHA (*sn*-3)**	**EPA (*sn*-2)/DHA (*sn*-3)**
CHCA1	52	2	12	4
CMBT1	0	0	0	0
DHB1	0	0	0	0
DHB2	12	0	12	0

* Matrices (CHCA1, CMBT1, DHB1, and DHB2) abbreviations as expressed in Section 4.7. EPA and DHA presence is highlighted in bold.

**Table 4:** FA composition and quantification (g/100 g TFA) of sAG extractions realized during lipase catalysis under CO_2_SC.

Fatty Acid	Extraction 1 (30 min)	Extraction 2 (60 min)	Extraction 3 (120 min)
12:0	0.07 ± 0.00	0.11 ± 0.00	0.13 ± 0.00
14:0	2.88 ± 0.01	3.66 ± 0.03	4.48 ± 0.02
16:0	12.65 ± 0.05	14.12 ± 0.03	14.82 ± 0.04
9t-16:1	0.18 ± 0.00	0.07 ± 0.00	0.10 ± 0.00
9c-16:1	3.82 ± 0.02	4.25 ± 0.04	4.61 ± 0.06
17:0	0.28 ± 0.00	0.22 ± 0.00	0.23 ± 0.00
10c-17:1	0.13 ± 0.00	0.14 ± 0.00	0.15 ± 0.00
18:0	3.59 ± 0.01	3.50 ± 0.01	3.21 ± 0.02
9c-18:1	35.95 ± 0.10	36.38 ± 0.06	35.80 ± 0.01
7c-18:1	4.28 ± 0.02	3.30 ± 0.07	3.40 ± 0.01
9c, 12c-18:2	15.80 ± 0.02	15.84 ± 0.00	16.13 ± 0.08
20:0	0.29 ± 0.00	0.27 ± 0.00	0.22 ± 0.00
6c, 9c, 12c-18:3	0.21 ± 0.00	0.23 ± 0.00	0.24 ± 0.00
8c-20:1	0.45 ± 0.01	0.45 ± 0.00	0.41 ± 0.00
11c-20:1	1.63 ± 0.00	1.63 ± 0.01	1.15 ± 0.01
9c, 12c, 15c-18:3	5.16 ± 0.03	4.84 ± 0.04	5.07 ± 0.03
11c, 14c-20:2	1.29 ± 0.01	1.27 ± 0.02	1.17 ± 0.00
22:0	0.32 ± 0.00	0.33 ± 0.00	0.31 ± 0.00
11c, 14c, 17c-20:3	0.60 ± 0.01	0.28 ± 0.00	0.27 ± 0.00
13c-22:1	0.30 ± 0.00	0.22 ± 0.00	0.17 ± 0.00
5c, 8c, 11c, 14c-20:4	0.33 ± 0.00	0.32 ± 0.00	0.31 ± 0.00
13c, 16c-22:2	0.11 ± 0.00	0.09 ± 0.00	0.07 ± 0.00
5c, 8c, 11c, 14c, 17c-20:5 *	3.82 ± 0.01 ^a^	3.68 ± 0.02 ^b^	3.57 ± 0.01 ^c^
9c-24:1	0.24 ± 0.00	0.22 ± 0.00	0.20 ± 0.00
7c, 11c, 13c, 16c-22:4	0.12 ± 0.00	0.11 ± 0.00	0.09 ± 0.00
7c, 10c, 13c, 16c, 19c-22:5	1.70 ± 0.00	1.36 ± 0.02	1.11 ± 0.00
4c, 7c, 10c, 13c, 16c, 19c-22:6 *	3.79 ± 0.00^a^	3.11 ± 0.01^b^	2.60 ± 0.00^c^

* Values ± standard deviation followed by different superscripts (a,b,c) denote significant (*p* < 0.05) differences.

**Table 5:** FAs identified with the greatest probability in Extraction 1 (30 min) in the CHCA1 matrix *.

*m/z*	MG and DG Identified in Extraction 1
379.2819	MG (18:1/–/–) MG (18:1/–/–), MG (–/18:1/–)
379.2843	MG (–/20:4/–)
587.5585	DG (–/16:0/18:1)
587.4646	DG (14:0/18:2/–), DG (16:1/16:1/–), DG (14:1/18:1/–), DG (12:0/20:2/–)
587.4670	DG (14:1/20:4/–), DG (12:0/22:5/–), DG (14:0/**20:5**/–), DG (18:4/16:1/–)
587.5221	DG (16:1/17:0/–), DG (14:1/19:0/–)

* EPA presence in *sn*-2 position is highlighted in bold. CHCA1 matrix abbreviation as expressed in Section 4.7.

**Table 6:** FAs identified with the greatest probability in Extraction 2 (60 min) in the CHCA1 matrix *.

*m/z*	MG and DG Identified in Extraction 2
379.2819	MG (18:1/–/–), MG (18:1/–/–), MG (–/18:1/–)
379.2843	MG (–/20:4/–)
603.4385	DG (14:0/18:2/–), DG (16:1/16:1/–), DG (15:1/17:1/–), DG (15:0/17:2/–), DG (14:1/18:1/–)
603.4385	DG (12:0/20:2/–)

* CHCA1 matrix abbreviation as expressed in Section 4.7.

**Table 7:** Presence of EPA or DHA in *sn*-2 position in the DG and TG of sAG identified with the greatest probability in Extraction 3 (120 min) in the CHCA1 matrix *.

*m/z*	MG and DG Identified in Extraction 3
377.2686	MG (–/**20:5**/–)
409.2925	MG (–/**22:6**/–)
441.2402	MG (–/**22:6**/–)
585.4514	DG (14:1/**20:5**/–), DG (12:0/**22:6**/–)
599.467	DG, 13:0/**22:6**/–), DG (15:1/**20:5**/–)
601.4827	DG (15:0/**20:5**/–)

* EPA and DHA presence in *sn*-2 position is highlighted in bold. CHCA1 matrix abbreviation as expressed in Section 4.7.

## Data Availability

Not applicable.

## Sample Availability

Samples of the compounds are not available from the authors.
